# ITHindex: An integrated web-based platform for intratumor heterogeneity evaluation

**DOI:** 10.1371/journal.pdig.0001654

**Published:** 2026-08-28

**Authors:** Yutao Liu, Yuan Jiang, Wenchuan Xie, Feidie Duan, Shuiting Fu, Jing Zhao, Jinyu Yang, Jianchun Duan, Guoqiang Wang, Yuzi Zhang, Shangli Cai, Yongqin Wen

**Affiliations:** 1 Department of Medical Oncology, State Key Laboratory of Molecular Oncology, National Cancer Center/National Clinical Research Center for Cancer/Cancer Hospital, Chinese Academy of Medical Sciences and Peking Union Medical College, Beijing, China; 2 The Affiliated Cancer Hospital of Xiangya School of Medicine, Central South University/Hunan Cancer Hospital, Changsha, Hunan, China; 3 Burning Rock Biotech, Guangzhou, Guangdong, China; 4 Department of Oral and Maxillofacial-Head Neck Oncology, Department of Laser and Aesthetic Medicine, Shanghai Ninth People’s Hospital, College of Stomatology, Shanghai Jiao Tong University School of Medicine, Shanghai, China; 5 Department of Pathology, Shenzhen University of Advanced Technology General Hospital, Shenzhen, Guangdong, China; Beth Israel Deaconess Medical Center, UNITED STATES OF AMERICA

## Abstract

Intratumor heterogeneity (ITH) is a critical factor influencing cancer progression, therapeutic response, and the development of drug resistance. Despite its importance, the lack of a definitive gold standard for ITH quantification has hindered consistent clinical application. To bridge this gap, we developed *ITHindex*, a web-based, research-enabling platform that integrates a pragmatic subset of 17 user-accessible ITH algorithms within the R Shiny framework. The tool supports diverse data modalities, including somatic mutation, copy number variation, transcriptomic, proteomic, and methylation profiles. By analyzing 11,242 samples across 32 cancer types from The Cancer Genome Atlas (TCGA) and 4,904 samples from cBioPortal, alongside 398 paired tissue and plasma samples, we validated the platform’s utility in quantifying ITH and characterizing the relationships between diverse metrics. *ITHindex* streamlines the computational pipeline, providing researchers with a robust tool for systematic ITH investigation and data-driven biomarker discovery. The *ITHindex* server is freely accessible at https://shinyapps.brbiotech.com/app/ithindex.

## Author summary

Prior knowledge indicated that ITH mirrors the complexity of tumor clones and holds promise as a predictive biomarker for the efficacy of immunotherapy. Several algorithms have been developed to quantify ITH based on omics data. However, implementing these algorithms often involves multiple software tools, advanced programming skills, and complex workflows, which can hinder accessibility and reproducibility. We introduced *ITHindex*, an integrated platform that integrated these algorithms to streamline the practice of ITH quantification for clinical researchers. *ITHindex* combines these algorithms into a user-friendly platform, seamlessly integrating computational methods with proprietary modules through an intuitive graphical interface. By integrating modality-specific algorithms, multi-omics data, and customizable parameters, the platform empowers researchers to conduct ITH quantification without coding. This platform reduces technical barriers, enhances reproducibility, and fosters collaborative, data-driven biomarker discovery.

## Introduction

While originating from a single rogue somatic cell, a tumor commonly evolves into a complex ecosystem consisting of billions of cells exhibiting significant genetic and non-genetic variations, a phenomenon referred to as intratumor heterogeneity (ITH) [1]. Beyond traditional genomic aberrations, ITH is increasingly recognized as a multidimensional phenomenon that profoundly influences the tumor microenvironment (TME) and interacts with the intratumoral microbiome [2,3]. These non-genomic layers of heterogeneity — manifest in cellular morphology, metabolism, immune infiltration, and microbial diversity — create distinct ecological niches that drive disease progression, immune evasion, and treatment resistance [4–6]. Consequently, understanding this multidimensional landscape is essential for developing effective cancer therapies By recognizing and integrating these diverse facets of ITH, researchers and clinicians can work towards personalized strategies that target specific characteristics of cancer subgroups, ultimately improving patient outcomes.

Numerous metrics have been developed to assess ITH using genetic, transcriptomic, proteomic, and epigenetic data. Major metrics for genetic ITH (gITH) include Jaccard Similarity index [7], Mutant-Allele Tumor Heterogeneity (MATH) score [8], EXPANDS [9], PhyloWGS [10], EstimateClonality [11], Shannon entropy [12], and Copy number heterogeneity (CNH/CNHplus) [13]. For transcriptomic ITH (tITH), specific metrics include network-based Jensen-Shannon Divergence (nJSD) [14], standard deviations (SD) of absolute z-scored transcriptome levels (DEPTH2) [15] and Shannon entropy [16]. Proteomic ITH (pITH) and epigenetic data (epiITH) have fewer dedicated metrics. Among these, MATH, as one of the earliest algorithms, has been demonstrated to predict poor prognosis in head and neck squamous cell carcinomas, uterine corpus endometrial carcinoma, lung adenocarcinoma, breast cancer, and colorectal cancer based on elevated MATH-derived ITH scores [17]. Similarly, other algorithms have also been validated for their role in predicting prognosis across different cancer types [17]. In addition to prognosis prediction, Shannon entropy-based blood ITH has revealed that patients with lower ITH metrics experience notable overall survival (OS) and progression-free survival (PFS) benefit from immune checkpoint blockade therapies compared with chemotherapy [18].

Despite the abundance of available metrics and the promise for guiding treatment in clinical settings, a significant challenge remains that there is often a marked discordance among different ITH algorithms [19]. Because these methodologies rely on distinct statistical foundations—ranging from allele frequency distribution to phylogenetic tree reconstruction—results can vary substantially even when analyzing the same patient cohort [17]. Such inconsistencies hinder the establishment of standardized clinical thresholds and limit the reproducibility of ITH-based biomarkers across different studies. Furthermore, the current metrics are formulated using a range of tools, including those in R language, MATLAB, and some lacking calculation scripts entirely. The lack of a unified analytical framework makes it difficult for researchers to perform systematic comparisons or cross-validation.

To address this gap, we developed *ITHindex*, an interactive platform designed to enhance methodological rigor in tumor heterogeneity research. Researchers can upload their omics data to *ITHindex* and select from a range of metrics to compute relevant ITH scores. Beyond mere computational convenience, *ITHindex* facilitates the acquisition of multi-algorithmic perspectives, enabling researchers to evaluate the comparability of various metrics and ensuring that biological conclusions are robust rather than algorithm-dependent. By integrating diverse omics layers into a standardized pipeline, *ITHindex* aims to bridge the gap between complex evolutionary theory and reproducible precision oncology.

## Results

### Overview of *ITHindex*

*ITHindex* is an open-access web platform developed to integrate a pragmatic subset of user-accessible algorithms for assessing ITH. The current version of *ITHindex* (v0.2.4) integrates a curated selection of 17 algorithms derived from 14 seminal publications, which were filtered through specific inclusion and exclusion criteria to provide a practical toolset of Level 3 data-compatible methods across genomic (n = 8), transcriptomic (n = 5), proteomic (n = 2), and epigenetic (n = 2) modalities (S1 Table, S2A Fig). In terms of the number of sampling regions, 3 metrics involved multi-region sampling, while the remaining articles focused on single-region sampling. Regarding sample processing for detection, one metric utilized single-cell sequencing, while the others employed bulk DNA or RNA detection methods. From the perspective of algorithmic principles, all metrics were categorized into two types: statistical (descriptive and spatial) and information theory. Statistical algorithms are based on various statistical measures or methods to evaluate ITH, including similarity measure, mean absolute deviation (MAD), ratio, standard deviation, distance measures, and principal component analysis (PCA). The algorithm, grounded in information theory principles, utilized Shannon entropy to calculate ITH. The final output results are contingent on users’ selections of omics data and calculation methodologies.

**Fig 1:**
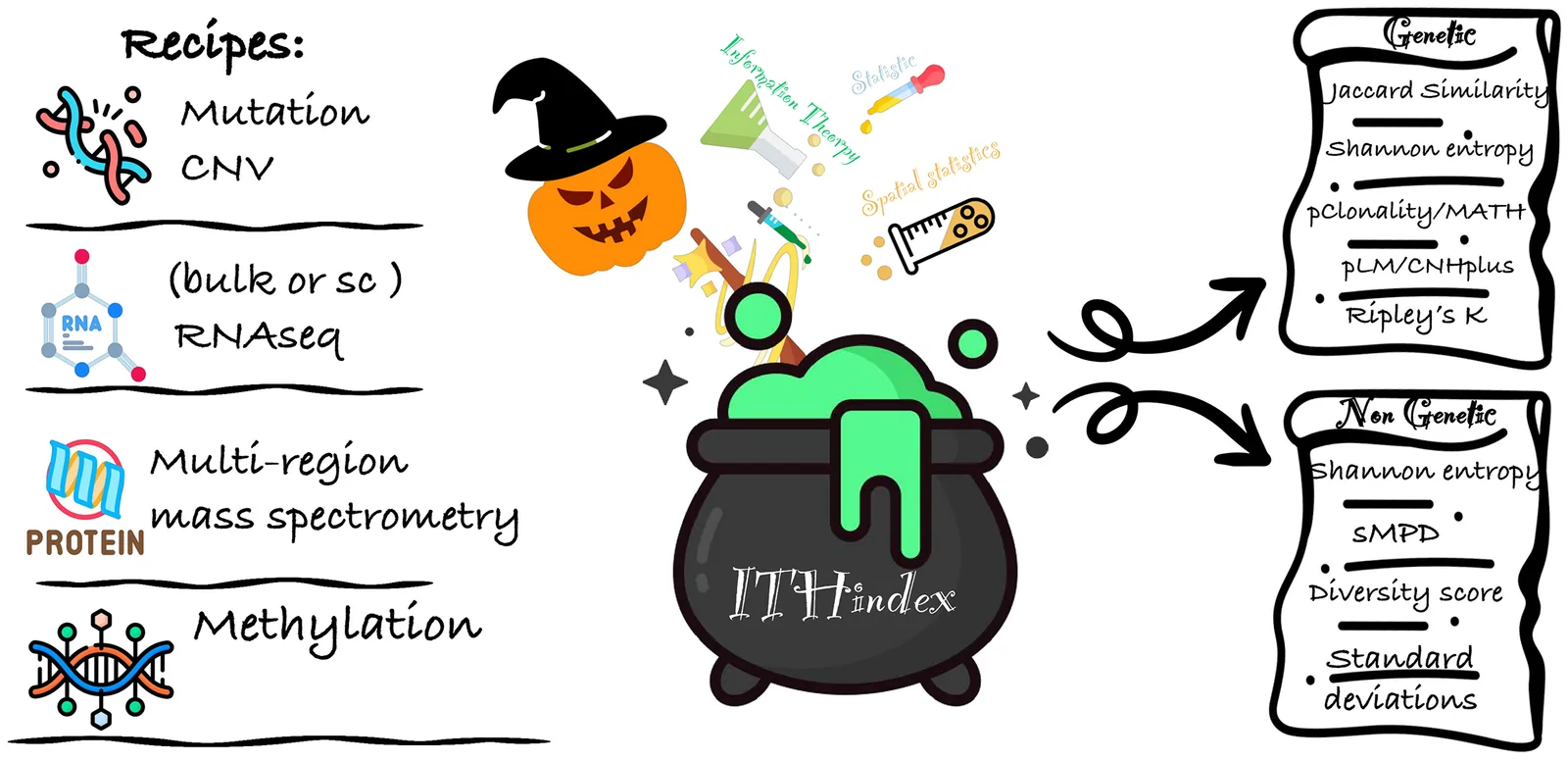
Overview of *ITHindex.*

*ITHindex* has already generated results for a total of 11,242 samples from TCGA utilizing 10 distinct metrics, which are downloadable as CSV files from the platform. Users can compute results for their own data, saving them locally in CSV format for subsequent research and analysis. Fig 1 provides an overview of the *ITHindex* tool’s framework.

### Workflow and functional modules of *ITHindex*

*ITHindex* is powered by Shiny and offers a wide range of options for quantifying ITH. We have created a user-friendly web interface with the aim of incorporating multiple algorithms for ITH quantification, which are accessible through four main pages: “Home”, “ITH Index”, “Data” and “About”. The “Home” page provides an overview of *ITHindex*; the “ITH Index” page allows users to upload and analyze their own data; the “Data” section offers pre-analyzed ITH data from TCGA samples; and the “About” page includes four sections: “About *ITHindex*”, “Contact Us”, “Acknowledgments” and “FAQ (Frequently Asked Questions)”.

**Fig 2:**
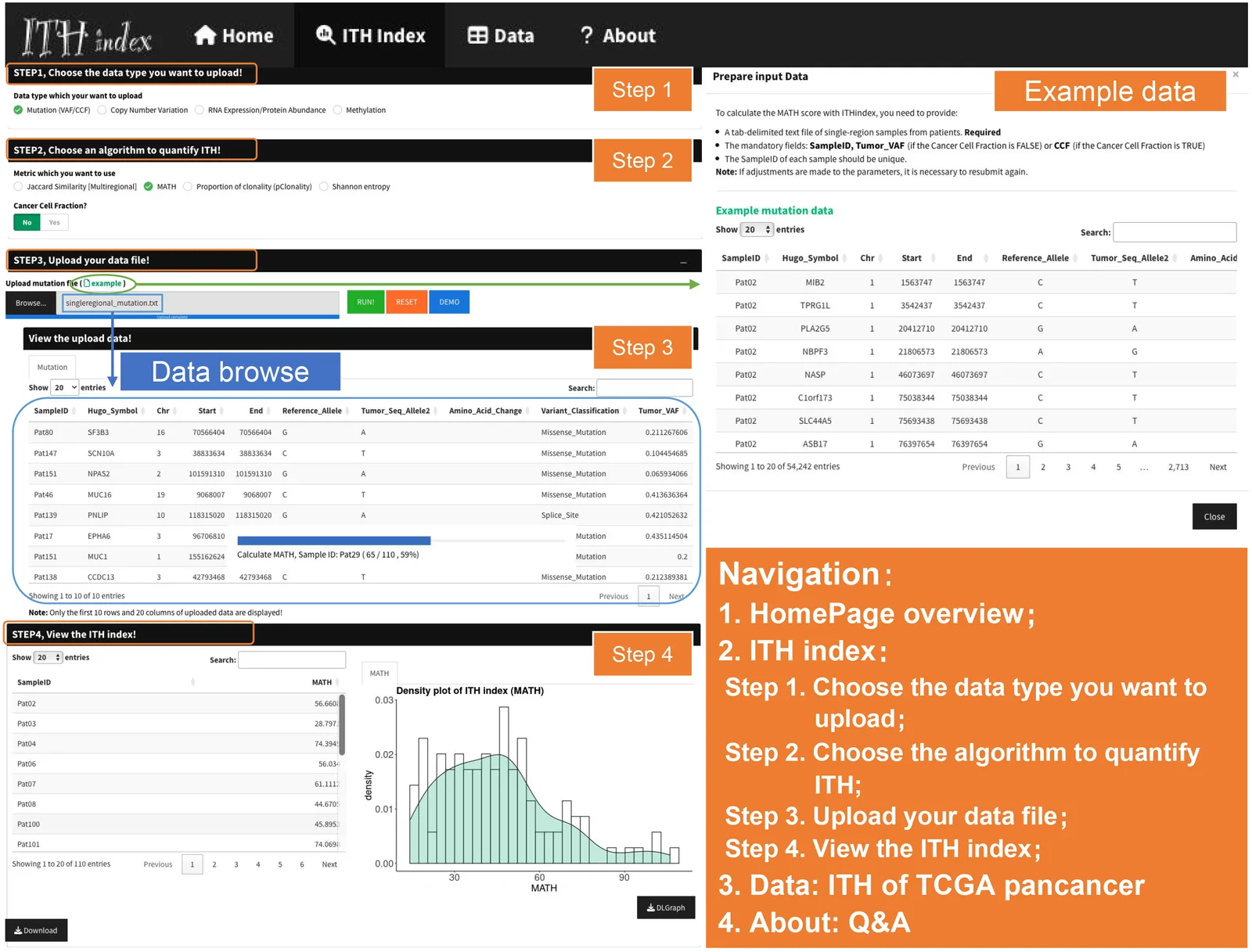
A standard analysis workflow for *ITHindex.*

On the “ITH Index” tab, the ITH calculation process is segmented into four steps within an interactive collapsible box. A well-defined analysis workflow is illustrated in Fig 2. Initially, users can select the data type from four options provided by *ITHindex*: mutation data with variant frequency (VAF) or cancer cell fraction (CCF), copy number variation, mRNA expression/protein abundance, and methylation. Subsequently, users may choose an algorithm for quantifying ITH, with the option to customize specific parameters for certain algorithms. For example, when selecting “Mutation (VAF/CCF)” and “Shannon entropy”, users can adjust parameters such as Cancer Cell Fraction (Yes/No), Number of bins (10–100), Adjusted by max VAF/CCF (Yes/No), and Weighted to entropy (Yes/No) to suit their requirements. Users can tailor these parameters according to their preferences. Next, users can upload their data file from their local device through the browser tab. To facilitate optimal utilization of the tool, example data files for each data type in every algorithm are provided, and users are strongly encouraged to follow these examples when uploading their data. Upon uploading the data file, the subsequent tab, “View the upload data” displays the initial 10 rows and 20 columns of the uploaded data for verification. Finally, the tool presents the results table and plots after clicking “RUN!” button. *ITHindex* offers downloadable results and density plots for user access.

To ensure the robustness and reproducibility of ITH quantification, *ITHindex* implements a multi-layer validation pipeline. First, upon data upload, the platform performs real-time checks for input integrity, such as verifying column headers, detecting missing values, and validating data format compatibility with the chosen algorithm. Second, to facilitate reproducibility, *ITHindex* generates an “Analysis Parameters” box accompanying the results. This tab captures a comprehensive snapshot of the session, including the specific algorithm applied, all user-defined parameters, and the timestamp of the analysis. This documentation ensures that each computational step is transparent and verifiable, mitigating the risk of inadvertent parameter drift across analyses.

### Comparison of various algorithms for ITH evaluation

To demonstrate the versatility and utility of *ITHindex*, we performed three comparative analyses using both public and in-house datasets.

#### Case 1: Cross-algorithm consistency and metric correlation.

A critical challenge in ITH research is determining whether outcome predictions are dependent on the choice of algorithm. *ITHindex* streamlines this by enabling rapid, multi-algorithm quantification.

**Fig 3:**
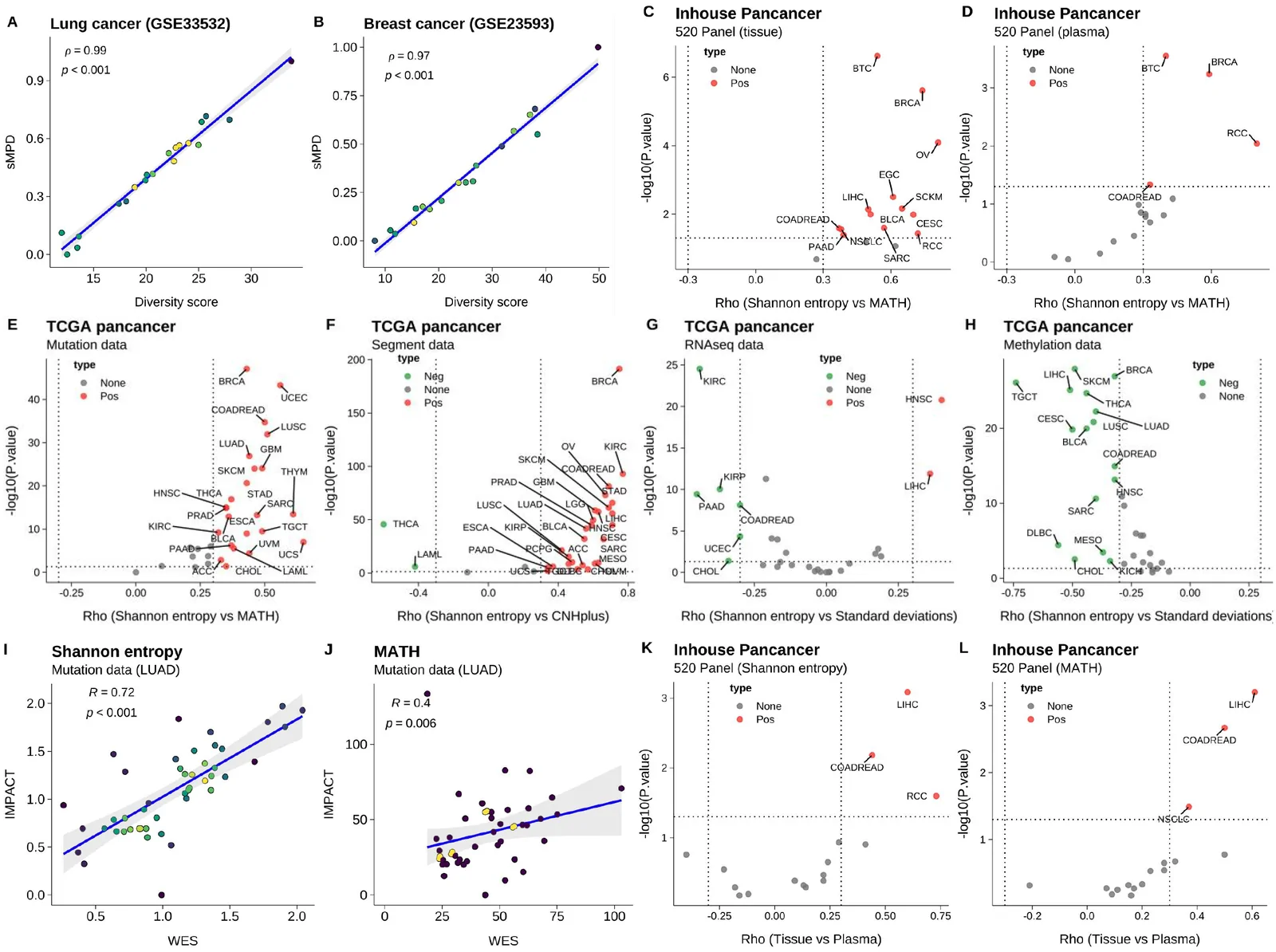
The correlation analyses with ITH scores obtained from *ITHindex.* **(A)** The correlation between sMPD and diversity score for the mRNA expression data.

First, we analyzed multi-regional bulk mRNA expression data from a lung cancer cohort (GSE33532). Using the two ITH metrics, scaled mean pairwise distance (sMPD) and diversity score, we identified a strong positive correlation (r = 0.99, P < 0.001; Fig 3A). Consistent results were observed across breast, liver, and cervical cancer datasets (Fig 3B, S2B-C Fig). The observed high degree of correlation is likely attributable to their shared theoretical foundations, as both algorithms are distance-based metrics designed to quantify divergence in expression profiles. Second, we compared Shannon entropy with MATH for mutation data across different sequencing strategies. Regardless of whether the data originated from targeted NGS panels or Whole-Exome Sequencing (WES), these two metrics demonstrated significant positive correlations in 13 of 16 cancer types (all P < 0.05, r > 0.3; Fig 3C-3E). Notably, however, for plasma-derived samples, significant correlations were only maintained in a subset of tumor types, specifically BRCA, RCC, and BTC (Fig 3D). Furthermore, we compared Shannon entropy with CNHplus using TCGA copy number variation (CNV) profiles. Our results showed that these two metrics were significantly correlated in most malignancies, encompassing 29 of 32 cancer types (all P values < 0.05, r > 0.3, Fig 3F). Last, when analyzing mRNA expression and methylation data, the correlation between Shannon entropy and SD-based ITH metrics was generally low, reaching significance only in HNSC and LIHC (Fig 3G-3H). These findings underscore that while algorithms with similar mathematical rationales yield highly concordant results, metrics derived from different omics layers or distinct statistical frameworks often capture divergent biological facets of ITH. This highlights the necessity of the multi-algorithmic approach provided by *ITHindex*.

in lung cancers; **(B)** The correlation between sMPD and diversity score for the mRNA expression data in breast cancers; **(C)** The correlation between Shannon entropy and MATH for mutation data derived from NGS panel data of tissue samples; **(D)** The correlation between Shannon entropy and MATH for mutation data derived from NGS panel data of plasma samples; **(E)** The correlation between Shannon entropy and MATH for tissue WES mutations data; **(F)** The correlation between Shannon entropy and CNHplus for tissue copy number data; **(G)** The correlation between Shannon entropy and standard deviations for tissue RNAseq data; **(H)** The correlation between Shannon entropy and standard deviations for tissue methylation data. **(I)** The ITH index correlations of Shannon entropy between WES and NGS panel; **(J)** The ITH index correlations of MATH between WES and NGS panel; **(K)** The ITH index correlations of data from tissue with plasma sample for Shannon entropy; **(L)** The ITH index correlations of data from tissue with plasma sample for MATH.

#### Case 2: Benchmarking NGS panels against WES.

To evaluate the reliability of the integrated algorithms, we conducted a systematic performance assessment focusing on platform heterogeneity, sequencing depth, and sample size. MATH and Shannon entropy were chosen as representative metrics for this validation due to their widespread application in clinical research [18,20–22]

First, we utilized 46 paired WES and NGS panel samples to evaluate the performance of Shannon entropy and MATH. The results showed the MATH-based ITH between the NGS panel and WES was lower than Shannon entropy (r of Shannon entropy: 0.72, P < 0.001, r for MATH: 0.40, P = 0.006, respectively, Fig 3I-3J). Second, genomic data from two LUAD cohorts were used to assess the stability of MATH and Shannon entropy across varied sequencing platforms and depths. As shown in Table 1 and S3 Fig, the ctDNA panel detected a wider range of VAFs (MAD: 0.009 for the ctDNA panel vs. 0.120 for WES, Table 1), and MATH-based ITH scores derived from the ctDNA panel were significantly higher than those from WES (median: 0.000 vs. 44.232, P < 0.05, S3 Fig). A consistent trend was observed in the comparison between the ctDNA panel and IMPACT (S3 Fig). Furthermore, although the MSK-IMPACT assay yielded a VAF MAD comparable to, or even higher than, that of WES (MAD: 0.156 for IMPACT vs. 0.132 for WES), the MATH score derived from IMPACT was significantly lower than that from WES (median: 39.508 vs. 49.773, P < 0.05). Our analysis reveals that as sequencing depth increases, the distribution of VAFs becomes more concentrated, evidenced by a significant reduction in the VAF MAD. Consequently, the MATH shows a significant decrease as sequencing depth increases (P < 0.05 for each comparison). While Shannon entropy displays a similar downward trend, it did not reach statistical significance in tissue samples when comparing WES and IMPACT panel sequencing (P = 0.260, S3 Fig). These findings suggest that depth-dependent VAF concentration exerts a stronger influence on MATH compared to Shannon entropy in high-coverage scenarios [23]. Last, to characterize the impact of sample size, we investigated the relationship between mutation data and ITH metrics using four tumor cohorts sequenced with the MSK-IMPACT panel and four same cancer types from TCGA cohorts (WES). We observed that both MATH and Shannon entropy exhibit minor fluctuations when the sample size is limited (n < 100) (S4 Fig). However, these fluctuations were consistent with the underlying shifts in tumor VAF distributions, indicating that the metrics remain stable and biologically coherent across diverse cohort sizes.

**Table 1:** The median and MAD of MATH and Shannon entropy across varied sequencing platforms.

Cohort	Platform	Sample Size	VAF		MATH		Entropy	
			MAD	Median	MAD	Median	MAD	Median
LUAD (CC2023)	IMPACT	77	0.156	0.235	18.359	39.508	0.216	0.331
LUAD (CC2023)	WES	77	0.132	0.188	16.385	49.773	0.182	0.296
LUAD (NM2022)	cfDNA_panel	265	0.031	0.025	5.495	3.707	0.000	0.000
LUAD (NM2022)	IMPACT	265	0.165	0.234	22.832	32.131	0.241	0.245
LUAD	cfDNA_panel	16	0.009	0.008	0.000	0.000	0.000	0.000
LUAD	WES	16	0.120	0.183	16.293	48.232	0.172	0.293

#### Case 3: Feasibility of plasma-based ITH assays.

To explore the potential of liquid biopsies in replacing tissue-based ITH assays, we analyzed 398 pancancer samples with paired tissue and plasma samples sequenced using identical NGS panels. While the correlation of MATH and Shannon entropy metrics between tissue and plasma was generally weak across most cancer types, distinct cohorts, including LIHC, CRC, RCC, and NSCLC showed significantly higher concordance (Fig 3K-L). This observed discordance suggests that the ITH profiles derived from tissue and plasma are likely driven by the distinct biological properties of the samples, specifically the localized spatial heterogeneity inherent in tissue biopsies versus the integrated systemic tumor burden captured by ctDNA, rather than technical biases or algorithmic limitations. To move beyond a theoretical concept and demonstrate the practical utility of this discordance, we introduced “Delta-ITH” (∆, defined as the tissue ITH score minus the plasma ITH score) as a novel dimension for understanding tumor evolution.

To rigorously validate this framework, we performed an in-depth clinical analysis on an independent cohort of 265 lung adenocarcinoma (LUAD, NM2022) patients who possessed paired tissue-plasma sequencing and complete clinical and follow-up data. Consistently, tissue-derived ITH metrics demonstrated a significant positive correlation with plasma-derived counterparts in this cohort (both MATH and Shannon entropy: R = 0.21, P < 0.001, S5A-B Fig). We next evaluated whether Delta-ITH correlated with basic demographic factors. While Delta-ITH did not significantly vary across age groups (<60 vs. ≥ 60 years), male patients displayed higher Delta-ITH values than female patients, which achieved statistical significance (∆-MATH: P = 0.092; ∆-Shannon entropy: P = 0.013, S5C-D Fig).

**Fig 4:**
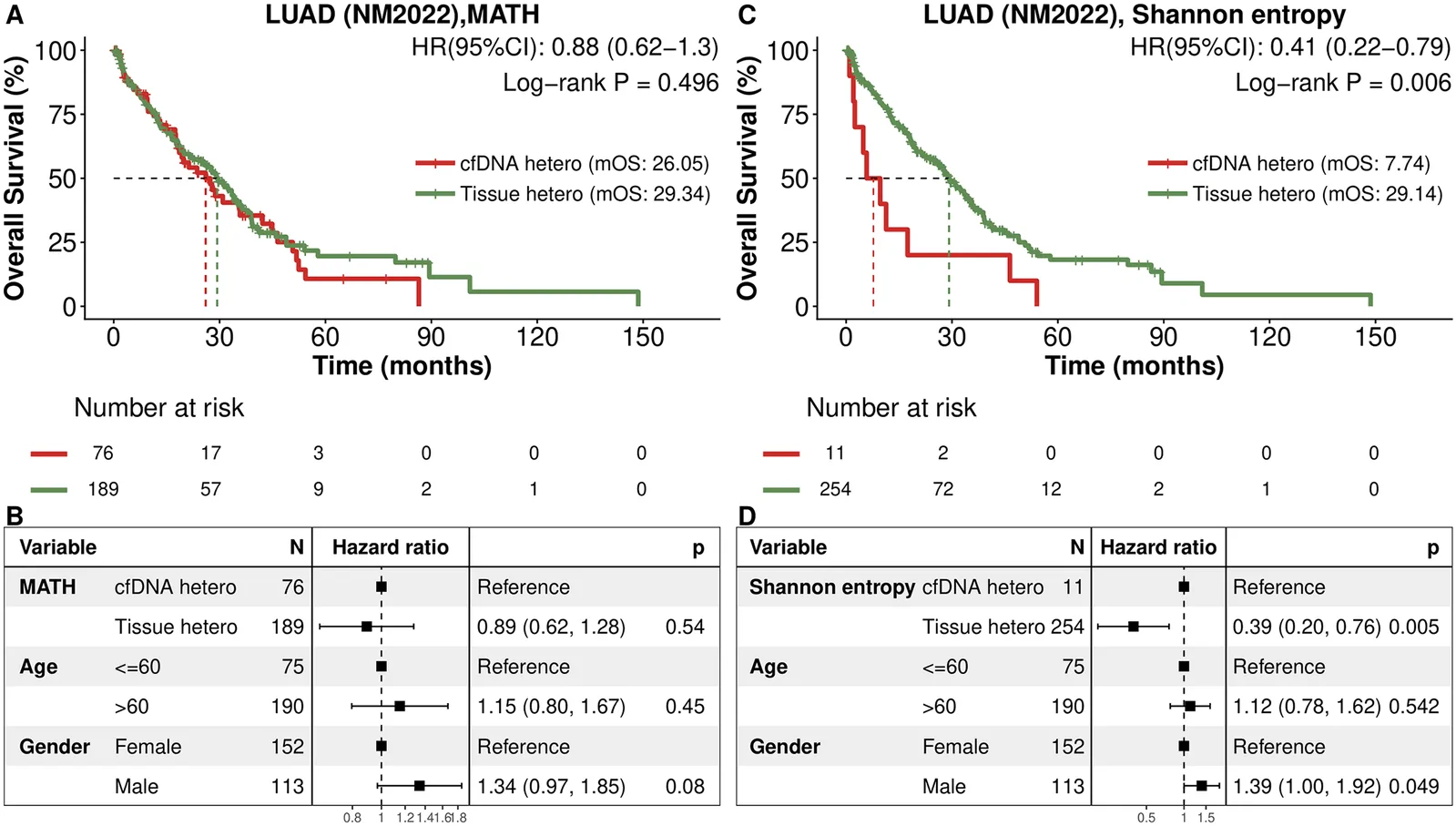
Prognostic Value of Delta-ITH. **(A)** Kaplan-Meier OS curves of LUAD patients stratified by MATH-based Delta-ITH; **(B)** Forest plot of multivariable Cox proportional hazards regression analysis evaluating the clinical independence of MATH-based Delta-ITH stratification alongside standard clinical covariates (age and gender); **(C)** Kaplan- Meier OS curves of LUAD patients stratified by Shannon entropy-based Delta-ITH; **(D)** Forest plot of multivariable Cox proportional hazards regression analysis evaluating the prognostic value of Shannon entropy-based Delta-ITH stratification.

Crucially, we investigated the prognostic value of Delta-ITH by stratifying patients into a tissue-heterogeneity predominant group (Tissue-hetero, ∆ ≥ 0) and a circulating-heterogeneity predominant group (cfDNA-hetero, ∆ < 0). While no significant survival difference was observed using the MATH-based Delta-ITH stratification (Fig 4A-B), survival analysis revealed that based on Shannon entropy, the cfDNA-hetero group experienced significantly worse OS compared to the Tissue-hetero group (HR = 0.41, 95%CI: 0.22-0.79, Log-rank P = 0.006, Fig 4C). Multivariable Cox regression confirmed that this prognostic value was independent of standard clinical factors (P = 0.005, Fig 4D).

Biologically, the poorer survival in the cfDNA-hetero population suggests that advanced tumor progression, often accompanied by accelerated cell apoptosis and necrosis, drives a higher systemic shedding of diverse subclonal DNA into the bloodstream. Consequently, plasma-derived Shannon entropy captures a more comprehensive, global tumor heterogeneity than a single localized tissue biopsy, aligning with established liquid biopsy paradigms where ctDNA dynamics reflect systemic tumor burden

Biologically, these findings suggest that in advanced-stage cancer patients, a higher volume of cfDNA is shed into the bloodstream. In this context, utilizing Shannon entropy on plasma samples captures global tumor heterogeneity more effectively than a single-site localized tissue biopsy. This observation aligns perfectly with methodological approaches adopted by previous hallmark liquid biopsy studies [18,20,24].

*ITHindex* supports the rapid benchmarking of novel algorithms. If a new algorithm is introduced, utilizing *ITHindex* to derive diverse ITH metric outcomes enables a rapid comparison of variances between the new algorithm and existing ones.

## Discussion

*ITHindex* is the first interactive and easily accessible web-based tool designed for oncologic researchers to analyze the ITH of cancer patients, which is associated with the diverse biological mechanisms and treatment responses. *ITHindex* enables users to upload their own data and offers a variety of algorithms based on mutation, copy number variations, mRNA expression, protein abundance, and methylation data. By providing this integrated interface, *ITHindex* aims to bridge the gap between high-dimensional omics data and ITH quantification, ultimately providing an integrated resource for the cancer research community.

Although ITH has been shown to have a profound impact on tumor progression, immune evasion, and treatment response in various cancer types [25,26], assessing heterogeneity in human tumor samples remains a significant challenge [27]. Numerous methodologies have been developed to evaluate different aspects of tumor heterogeneity. However, current metrics are often empirical, challenging to interpret, and difficult to integrate into a unified framework, thereby limiting their reproducibility and adoption in clinical settings. In this study, we have presented several examples to illustrate how *ITHindex* can support ITH-related research. *ITHindex* enables the rapid acquisition of ITH scores from various algorithms, facilitating subsequent analysis. It is important to emphasize that *ITHindex* is designed as a research-enabling, exploratory platform. While our results demonstrate the platform’s potential in facilitating ITH investigations, the tool is not intended for clinical decision-making or diagnosis.

*ITHindex* can also assist in constructing NGS panel-based tissue ITH or blood ITH to assess ITH consistency, or in comparing new and existing algorithms to evaluate the performance attributes of the new algorithm.

Variations in ITH metric performance across sequencing platforms underscore the critical influence of sequencing depth and detection sensitivity on subclonal architecture quantification [28]. Our comparative analysis reveals that while deep sequencing (e.g., ctDNA panels at ~10,000x) captures a broader spectrum of low-frequency variants—thereby altering the VAF distribution profile—this does not uniformly translate into inflated MATH scores. This indicates that MATH is not merely a function of VAF dispersion (MAD) but is also heavily modulated by the median VAF of the sample [8]. In lower-depth WES data, the lack of sensitivity to the “long tail” of low-frequency subclonal variants shifts the median VAF upward and may lead to overestimation of heterogeneity due to sampling noise. In contrast, higher-depth platforms provide a more granular resolution of the subclonal landscape, resulting in a more robust and biologically representative ITH estimate.

Comparing tissue-derived and plasma-derived ITH metrics provides a unique window into tumor biology, reflecting the spatial and systemic dimensions of heterogeneity [29]. Our findings suggest that in the context of controlled sequencing depth, the weak correlation between tissue and plasma ITH is likely a biological signal rather than a technical artifact. Tissue samples, obtained via focal biopsy, reflect localized spatial heterogeneity within a specific tumor region. In contrast, ctDNA acts as a ‘liquid biopsy’ that integrates clonal information from the entire tumor burden, including primary lesions, multifocal tumors, and distant metastases [30]. Consequently, the difference between these two metrics—which we term “Delta-ITH”—may provide clinical value beyond individual measurements. By providing a unified platform to quantify ITH from both tissue and plasma, *ITHindex* empowers researchers to explore this Delta-ITH, offering a more nuanced understanding of how localized spatial heterogeneity contributes to systemic tumor evolution and treatment response. We explicitly note that the Case 3 verification cohort was derived from the LAVA database. This database comprises fully anonymized and de-identified data and has been utilized in several peer-reviewed studies [31–34]. Although this proprietary database offered unique deep-sequencing validation, we underscore that *ITHindex* remains a completely independent, vendor-neutral application designed to process standard Level 3 data from any user-provided clinical or public repository.

While *ITHindex* provides a unified and streamlined platform for the systematic evaluation of ITH, several limitations should be noted. First, the current framework is primarily tailored for bulk sequencing, containing only a single algorithm for single-cell data. Second, the metrics are inherently sensitive to upstream data preprocessing and technical covariates (e.g., tumor purity,sequencing depth and normalization methods) [28]. Because *ITHindex* focuses on Level 3 data to remain highly accessible for biomedical researchers without advanced coding skills, it does not integrate raw-data-based tools like *epihet* [35], or *PhyloWG* [10]. We therefore strongly recommend users adopt standardized workflows like GATK Best Practices [36,37] to ensure cross-study comparability. Third, while we comprehensively validated the cross--platform and cross-sample robustness of MATH and Shannon entropy-owing to their widespread adoption and minimal input barriers--the baseline technical dependencies for the remaining fifteen algorithms remain unestablished. This is primarily constrained by the extreme scarcity of public clinical cohorts that simultaneously provide matched multi-platform data for copy number, transcriptomic, proteomic, and epigenetic modalities. Users should thus interpret cross-platform comparisons for these extended metrics with caution. Additionally, the cross-omics application of certain algorithms -- such as applying sMPD to bulk transcriptomics or Shannon entropy to bulk proteomics -- remains exploratory and requires further methodological validation. Fourth, the Delta-ITH analysis serves as a preliminary, exploratory framework, as gene coverage and sequencing depths inherently vary between tissue and blood panels. Finally, while *ITHindex* is intended primarily as a research-enabling, exploratory tool for hypothesis generation, its current lack of integrated clinical phenotype data may limit its immediate utility in exploring complex clinical studies. Future upgrades will continuously incorporate newly published algorithms, accommodate larger multi-center cohorts with rigorous statistical adjustments, and bridge ITH metrics with comprehensive clinical metadata to enhance immediate translational utility.

The clinical translation of ITH as a robust biomarker faces significant challenges, particularly regarding the standardization of analytical pipelines and the reproducibility of results across different sequencing platforms. Furthermore, the biological significance of ITH is highly context-dependent, showing distinct evolutionary characteristics across various tumor types. In the realm of precision medicine, assessing ITH dynamics—especially through longitudinal liquid biopsies—offers critical value for predicting responses to immunotherapy and targeted therapy, as subclonal diversity often drives immune evasion and acquired resistance [38].

Moving forward, the integration of heterogeneity information across genomic, transcriptomic, and epigenomic layers represents a significant frontier for comprehensive tumor characterization. While *ITHindex* currently evaluates different data types independently, multi-omics integration can provide deeper insights into the evolutionary dynamics between genetic alterations and adaptive phenotypic changes. However, achieving this integration presents substantial technical challenges, including the need for standardized statistical frameworks to normalize diverse data scales and the requirement for matched multi-omics samples from the same biological regions. Future upgrades of *ITHindex* will explore these integrative methodologies, potentially utilizing advanced systems biology approaches to bridge the gap between distinct omics layers and provide a more holistic view of ITH.

In conclusion, *ITHindex* is an intuitive, registration-free, and easy-to-use analysis tool that can meet the needs of oncologic researchers in exploring the ITH based on multi-omics data using users’ own data. We believe that *ITHindex* will become a stable and reliable oncological tool with ongoing refinements.

## Materials and methods

### ITH algorithm identification and selection criteria

To ensure a systematic and transparent selection of the ITH quantification algorithms integrated into *ITHindex*, we performed a structured literature search across PubMed and Web of Science using the search string: *(“intratumor heterogeneity” OR “intra-tumor heterogeneity” OR “ITH”) AND (“algorithm” OR “quantification” OR “metric” OR “measure”).* The search was conducted from Jan 1990 to Dec 2025. The identification and screening process followed the Preferred Reporting Items for Systematic Reviews and Meta-Analyses (PRISMA) guidelines, as illustrated in S1 Fig.

**Inclusion criteria:**

1. **Peer-Reviewed Validation:** Algorithms must be supported by peer-reviewed literature, either as primary method development publications or through documented application in high-impact studies.
2. **Standardized Data Compatibility:** Metrics must be compatible with Level 3 “processed” omics data (e.g., mutation, expression matrices, methylation beta values), ensuring accessibility for researchers without high-performance computing infrastructure for raw data processing.
3. **Algorithmic Transparency & Reproducibility:** Algorithms must provide sufficient methodological documentation, including detailed step-by-step mathematical formulations, pseudocode, or accessible open-source implementations (e.g., R, Python, MATLAB), allowing for robust integration into our automated pipeline.

**Exclusion Criteria:**

1. **Lack of Validation**: Metrics or algorithms lacking documentation in peer-reviewed journals.
2. **Requirement for Raw Data**: Algorithms strictly requiring raw data formats (e.g., FASTQ, BAM) without options for processing Level 3 data.
3. **Methodological Opacity**: Algorithms with proprietary implementations, inaccessible source code, or insufficient documentation.

### Pan-cancer data collection

We gathered human tumor-related data from three major publicly available databases: The Cancer Genome Atlas (TCGA), Gene Expression Omnibus (GEO) (https://www.ncbi.nlm.nih.gov/geo/), and cBioPortal (https://www.cbioportal.org/). Specifically, we obtained clinical information, WES data, copy number (CN) segments, RNA sequencing (RNAseq), and DNA methylation 27K microarray data for 32 tumor types from the TCGA database through cBioPortal. Additionally, we collected bulk expression profiles from four tumor types with multi-region sampling from the GEO database, including lung cancer (GSE33532) [39], breast cancer (GSE23593) [40], liver cancer (GSE136711) [41], and cervical cancer (GSE5787) [42]. Among these, GSE136711 provides RNAseq data, while the other three datasets offer mRNA microarray data. Additionally, five cohorts were retrieved from cBioPortal: three MSK-IMPACT targeted panel cohorts, including CRC (Gastro2020) [43], HCC (CCR2024) [44], and BRCA (CC2018) [45]; and two LUAD cohorts — LUAD (NM2022) and LUAD (CC2023) [46,47] — which encompass multi-platform sequencing data, such as tissue WES (~150x), tissue panels (MSK-IMPACT, ~ 800x), and plasma panels (~10,000x). Finally, we included in-house anonymization data from 398 patients who underwent next-generation sequencing (NGS) of a 520-gene panel (~10000x) on tumor DNA derived from both plasma and tissue samples, encompassing 16 types of solid tumors from the Burning Rock LAVA database (Burning Rock Biotech, Guangzhou, China). Altogether, 11,242 TCGA pan-cancer samples, 4,904 cBioPortal pan-cancer samples, and 398 in-house cases were included in this study. Detailed cohort information is provided in S2 Table.

### Application implementation

*ITHindex* was developed using the Shiny framework, enabling interactive data analysis and visualization with R. It leverages the tidyverse family of R packages (e.g., dplyr, purrr, and tibble) for internal tabular data transformations, the DT package for interactive data tables, and the ggplot2 and plotly packages for visualizing the ITH index. Static plots are created using ggplot2, while interactive plots are generated with the plotly package.

*ITHindex* can be deployed on the web server provided by shinyapps.io or on a local virtual machine using the ShinyProxy framework (https://www.shinyproxy.io), which organizes and manages Shiny application instances using Docker containers based on user demand. As a result, installation of R and RStudio on the user’s device is not required. The web-based tool is accessible via the following links: https://shinyapps.brbiotech.com/app/ithindex or https://xwen.shinyapps.io/ithindex/. Users can access the analysis tool through any standard browser, including Firefox, Chrome, Safari, or Microsoft Edge.

*ITHindex* is hosted on a server equipped with an 8-core CPU and 32 GB RAM to ensure robust computational performance and scalability. For a standard clinical cohort (e.g., 200 samples), the platform typically completes ITH quantification across multiple algorithms within 10 seconds, depending on the computational complexity of the selected metrics. To maintain infrastructure stability during user access, *ITHindex* incorporates proactive data validation, including format checks (e.g., file extension, column naming, and data type consistency) to identify malformed inputs before analysis. Furthermore, the web server currently supports a maximum upload file size of 50 MB per session, which accommodates the majority of processed “Level 3” multi-omics datasets. For larger pan-cancer datasets, we recommend that users upload data in smaller batches to adhere to file size constraints while maintaining optimal processing speeds.

To ensure long-term sustainability, *ITHindex* utilizes a structured maintenance strategy, with source code publicly hosted on GitHub under semantic versioning for transparent update tracking. Infrastructure stability is guaranteed by institutional support, which provides dedicated server resources for continued accessibility. We are committed to maintaining the web deployment for a minimum of five years post-publication, supported by an email-driven issue-tracking mechanism to facilitate ongoing user feedback and collaborative enhancement.

## Supporting information

S1 Fig PRISMA flow diagram for the literature search. (TIF)

S2 Fig ITH metrics and relationships. A. Collected ITH metrics, organized by type of heterogeneity, mathematical theory, and data type. B-C. The correlation between sMPD and diversity score for the same data type in HCC (B) and Glioma cancers (C).
(TIFF)

S3 Fig ITH metric divergence across varied sequencing platforms. A. Comparative analysis of MATH and Shannon entropy between MSK-IMPACT and WES in paired LUAD patients; B. Comparative analysis of MATH and Shannon entropy between cfDNA panel and MSK-IMPACT in paired LUAD patients; C. Comparative analysis of MATH and Shannon entropy between cfDNA panel and WES in paired LUAD patients.
(TIFF)

S4 Fig The trends of ITH metrics in relation to sample size across multiple cancer cohorts. A. CRC; B. HCC; C. BRCA; D. LUAD.
(TIFF)

S5 Fig Comparison of tissue and plasma ITH metrics and clinical association of Delta-ITH in LUAD patients. (A, B) Correlation analysis of (A) MATH and (B) Shannon entropy between paired tissue and plasma samples in LUAD patients. (C, D) Boxplots showing the association of (C) MATH-based and (D) Shannon entropy-based Delta-ITH with clinical characteristics (age and gender).
(TIFF)

S1 Table Collected intra-tumor heterogeneity metrics, organized by type of heterogeneity, mathematical theory, and data type. (DOCX)

S2 Table Detailed information of cohort used in this study. (DOCX)
