# Supplementary material for: ITHindex: An integrated web-based platform for intratumor heterogeneity evaluation
Source: PLOS Digit Health. 2026 Aug 28;5(8):e0001654. doi: 10.1371/journal.pdig.0001654 (PMC13524247; doi:10.1371/journal.pdig.0001654)
Supplement: Supplementary file 1 [file pdig.0001654.s006.docx]

**Online Supplementary Material**

Liu et al. ITHindex: An integrated web-based platform for intratumor heterogeneity evaluation

**Contents:**

**Supplementary Table 1**

**Table legends**

**Supplementary Table 1. Collected intra-tumor heterogeneity metrics, organized by type of heterogeneity, mathematical theory and data type**

| HeterogeneityType | Metric | Theory | InputDataType | Sampling | Reference |
| --- | --- | --- | --- | --- | --- |
| Genetic | Jaccard Similarity | Statistic | Mutation | Multiregional | PMID: 28092682 |
| Genetic | MATH | Statistic | Mutation | Single regional | PMID: 30341162 |
| Genetic | Proportion of clonality (pClonality) | Statistic | Mutation | Single regional | PMID: 33622313 |
| Genetic | Shannon entropy | information theory | Mutation | Single regional | PMID: 35902848 |
| Genetic | Proportion of late-stage mutations (pLM) | Statistic | Mutation and Segment | Single regional | PMID: 25877892 |
| Genetic | CNHplus | Statistic | Segment | Single regional | CNHplus: the chromosomal copy number heterogeneity which respects biological constraints |
| Genetic | Shannon entropy | information theory | Segment | Single regional | PMID: 28607403 |
| Genetic | Ripley’s K | Spatial statistics | Segment | Single regional | PMID: 28607403 |
| Transcriptomic | Standard deviations | Statistic | bulkRNAseq | Single regional | PMID: 35365157 |
| Transcriptomic | Shannon entropy | information theory | bulkRNAseq | Single regional | PMID: 18606989 |
| Transcriptomic | Scaled mean pairwise distance (sMPD) | Statistic | bulkRNAseq | Multiregional | PMID: 36419822 |
| Transcriptomic | Diversity score | Statistic | bulkRNAseq | Multiregional | PMID: 36527145 |
| Transcriptomic | Diversity score | Statistic | scRNAseq | Single regional | PMID: 31588021 |
| Proteomic | Shannon entropy | information theory | Proteome | Single regional | PMID: 18606989 |
| Proteomic | Scaled mean pairwise distance (sMPD) | Statistic | Proteome | Multiregional | PMID: 36419822 |
| Epigenetic | Standard deviations | Statistic | Methylation | Single regional | PMID: 34709741 |
| Epigenetic | Shannon entropy | information theory | Methylation | Single regional | PMID: 23177740 |
