# Supplementary material for: ITHindex: An integrated web-based platform for intratumor heterogeneity evaluation
Source: PLOS Digit Health. 2026 Aug 28;5(8):e0001654. doi: 10.1371/journal.pdig.0001654 (PMC13524247; doi:10.1371/journal.pdig.0001654)
Supplement: Supplementary file 2 [file pdig.0001654.s007.docx]

**Online Supplementary Material**

Liu et al. ITHindex: An integrated web-based platform for intratumor heterogeneity evaluation

**Contents:**

**Supplementary Table 2**

**Table legends**

**Supplementary Table 2. Detailed information of cohort used in this study**

| Source | Data type | Cohort name | Cancer type | Caner description | Num.Sample.Tissue | Num.Sample.Plasma |
| --- | --- | --- | --- | --- | --- | --- |
| Inhouse | NGS-520Panel | BLCA | BLCA | Bladder Urothelial Carcinoma | 24 | 24 |
| Inhouse | NGS-520Panel | BRCA | BRCA | Breast invasive carcinoma | 30 | 30 |
| Inhouse | NGS-520Panel | BTC | BTC | Biliary tract carcinoma | 80 | 80 |
| Inhouse | NGS-520Panel | CESC | CESC | Cervical squamous cell carcinoma and endocervical adenocarcinoma | 13 | 13 |
| Inhouse | NGS-520Panel | COADREAD | COADREAD | Colorectal adenocarcinoma | 36 | 36 |
| Inhouse | NGS-520Panel | EGC | EGC | Esophagogastric carcinoma | 21 | 21 |
| Inhouse | NGS-520Panel | HNSC | HNSC | Head and Neck squamous cell carcinoma | 23 | 23 |
| Inhouse | NGS-520Panel | LIHC | LIHC | Liver hepatocellular carcinoma | 28 | 28 |
| Inhouse | NGS-520Panel | NSCLC | NSCLC | Non-Small-Cell Lung Cancer | 34 | 34 |
| Inhouse | NGS-520Panel | OV | OV | Ovarian serous cystadenocarcinoma | 17 | 17 |
| Inhouse | NGS-520Panel | PAAD | PAAD | Pancreatic adenocarcinoma | 28 | 28 |
| Inhouse | NGS-520Panel | PRAD | PRAD | Prostate adenocarcinoma | 15 | 15 |
| Inhouse | NGS-520Panel | RCC | RCC | Renal cell carcinoma | 9 | 9 |
| Inhouse | NGS-520Panel | SARC | SARC | Sarcoma | 15 | 15 |
| Inhouse | NGS-520Panel | SCKM | SCKM | Skin Cutaneous Melanoma | 16 | 16 |
| Inhouse | NGS-520Panel | UCEC | UCEC | Uterine Corpus Endometrial Carcinoma | 9 | 9 |
| GEO | Microarray | GSE33532 | Lung cancer |  | 80 |  |
| GEO | Microarray | GSE23593 | Breast cancer |  | 50 |  |
| GEO | RNAseq | GSE136711 | HCC | Hepatocellular carcinoma | 41 |  |
| GEO | Microarray | GSE5787 | Cervical cancer |  | 33 |  |
| cBioPortal | MSK-IMPACT | luad_mskcc_2023_met_organotropism | LUAD | Lung adenocarcinoma | 1541 |  |
| cBioPortal | MSK-IMPACT | nsclc_ctdx_msk_2022 | LUAD | Lung adenocarcinoma |  | 733 |
| cBioPortal | MSK-IMPACT | hcc_msk_2024 | HCC | Hepatocellular carcinoma | 1294 |  |
| cBioPortal | MSK-IMPACT | crc_apc_impact_2020 | CRC | Colorectal adenocarcinoma | 467 |  |
| cBioPortal | MSK-IMPACT | breast_msk_2018 | BRCA | Breast invasive carcinoma | 869 |  |
| TCGA | WES/CNV/RNAseq/Methylation | ACC | ACC | Adrenocortical carcinoma | 91 |  |
| TCGA | WES/CNV/RNAseq/Methylation | BLCA | BLCA | Bladder Urothelial Carcinoma | 409 |  |
| TCGA | WES/CNV/RNAseq/Methylation | BRCA | BRCA | Breast invasive carcinoma | 1009 |  |
| TCGA | WES/CNV/RNAseq/Methylation | CESC | CESC | Cervical squamous cell carcinoma and endocervical adenocarcinoma | 281 |  |
| TCGA | WES/CNV/RNAseq/Methylation | CHOL | CHOL | Cholangiocarcinoma | 36 |  |
| TCGA | WES/CNV/RNAseq/Methylation | COADREAD | COADREAD | Colorectal adenocarcinoma | 528 |  |
| TCGA | WES/CNV/RNAseq/Methylation | DLBC | DLBC | Lymphoid Neoplasm Diffuse Large B-cell Lymphoma | 37 |  |
| TCGA | WES/CNV/RNAseq/Methylation | ESCA | ESCA | Esophageal carcinoma | 182 |  |
| TCGA | WES/CNV/RNAseq/Methylation | GBM | GBM | Glioblastoma multiforme | 395 |  |
| TCGA | WES/CNV/RNAseq/Methylation | HNSC | HNSC | Head and Neck squamous cell carcinoma | 502 |  |
| TCGA | WES/CNV/RNAseq/Methylation | KICH | KICH | Kidney Chromophobe | 65 |  |
| TCGA | WES/CNV/RNAseq/Methylation | KIRC | KIRC | Kidney renal clear cell carcinoma | 356 |  |
| TCGA | WES/CNV/RNAseq/Methylation | KIRP | KIRP | Kidney renal papillary cell carcinoma | 274 |  |
| TCGA | WES/CNV/RNAseq/Methylation | LAML | LAML | Acute Myeloid Leukemia | 140 |  |
| TCGA | WES/CNV/RNAseq/Methylation | LGG | LGG | Brain Lower Grade Glioma | 510 |  |
| TCGA | WES/CNV/RNAseq/Methylation | LIHC | LIHC | Liver hepatocellular carcinoma | 358 |  |
| TCGA | WES/CNV/RNAseq/Methylation | LUAD | LUAD | Lung adenocarcinoma | 562 |  |
| TCGA | WES/CNV/RNAseq/Methylation | LUSC | LUSC | Lung squamous cell carcinoma | 469 |  |
| TCGA | WES/CNV/RNAseq/Methylation | MESO | MESO | Mesothelioma | 82 |  |
| TCGA | WES/CNV/RNAseq/Methylation | OV | OV | Ovarian serous cystadenocarcinoma | 409 |  |
| TCGA | WES/CNV/RNAseq/Methylation | PAAD | PAAD | Pancreatic adenocarcinoma | 176 |  |
| TCGA | WES/CNV/RNAseq/Methylation | PCPG | PCPG | Pheochromocytoma and Paraganglioma | 178 |  |
| TCGA | WES/CNV/RNAseq/Methylation | PRAD | PRAD | Prostate adenocarcinoma | 493 |  |
| TCGA | WES/CNV/RNAseq/Methylation | SARC | SARC | Sarcoma | 234 |  |
| TCGA | WES/CNV/RNAseq/Methylation | SKCM | SKCM | Skin Cutaneous Melanoma | 440 |  |
| TCGA | WES/CNV/RNAseq/Methylation | STAD | STAD | Stomach adenocarcinoma | 436 |  |
| TCGA | WES/CNV/RNAseq/Methylation | TGCT | TGCT | Testicular Germ Cell Tumors | 144 |  |
| TCGA | WES/CNV/RNAseq/Methylation | THCA | THCA | Thyroid carcinoma | 485 |  |
| TCGA | WES/CNV/RNAseq/Methylation | THYM | THYM | Thymoma | 123 |  |
| TCGA | WES/CNV/RNAseq/Methylation | UCEC | UCEC | Uterine Corpus Endometrial Carcinoma | 515 |  |
| TCGA | WES/CNV/RNAseq/Methylation | UCS | UCS | Uterine Carcinosarcoma | 57 |  |
| TCGA | WES/CNV/RNAseq/Methylation | UVM | UVM | Uveal Melanoma | 80 |  |
